# [Expression of Concern] FHOD1 is upregulated in gastric cancer and promotes the proliferation and invasion of gastric cancer cells

**DOI:** 10.3892/ol.2026.15632

**Published:** 2026-04-29

**Authors:** Chengfei Jiang, Binbin Yuan, Bo Hang, Jian-Hua Mao, Xiaoping Zou, Pin Wang

Oncol Lett 22: 712, 2021; DOI: 10.3892/ol.2021.12973

Following the publication of the above paper, it has been drawn to the Editor's attention by a concerned reader that, regarding the cell invasion assay data shown in Fig. 2A on p. 7, two pairs of data panels shown for the shFHOD1 and LV-FHOD1 experiments contained overlapping sections, suggesting that these data panels, which were intended to show the results of differently performed experiments, had been derived from the same original source. A subsequent investigation of the data in this paper undertaken by the Editorial Office has revealed that a pair of the Control data panels in Fig. 8B also contain an overlapping section.

The authors have been contacted by the Editorial Office to offer an explanation for these apparent anomalies in the presentation of the data in this paper, and we are awaiting their response. Owing to the fact that the Editorial Office has been made aware of potential issues surrounding the scientific integrity of this paper, we are issuing an Expression of Concern to notify readers of this potential problem while the Editorial Office continues to investigate this matter further.

